# Identification of Anoikis-related potential biomarkers and therapeutic drugs in chronic thromboembolic pulmonary hypertension via bioinformatics analysis and in vitro experiment

**DOI:** 10.1038/s41598-024-75251-1

**Published:** 2024-12-28

**Authors:** Haijia Yu, Huihui Song, Jingchao Li, Luqian Cui, Shujuan Dong, Yingjie Chu, Lijie Qin

**Affiliations:** 1 Department of Emergency, Henan Provincial People’s Hospital, Zhengzhou, Henan China; 2 Department of Cardiology, Henan Provincial People’s Hospital, Zhengzhou, Henan China; 3 Department of Cardiac Care Unit, Henan Provincial People’s Hospital, Zhengzhou, Henan China

**Keywords:** CTEPH, Anoikis, Diagnostic markers, Therapeutic drugs, Immune infiltration, Machine learning, DNA, Computational biology and bioinformatics, Genetics, Biomarkers, Cardiology, Risk factors

## Abstract

There is growing evidence that programmed cell death plays a significant role in the pathogenesis of chronic thromboembolic pulmonary hypertension (CTEPH). Anoikis is a newly discovered type of programmed death and has garnered great attention. However, the precise involvement of Anoikis in the progression of CTEPH remains poorly understood. The goal of this study was to identify Anoikis-related genes (ARGs) and explore potential therapeutic drugs for CTEPH. Differentially expressed genes were identified by limma and weighted gene co-expression network analysis (WGCNA) packages, and functional analyses were conducted based on the differentially expressed genes. Subsequently, a combination of protein–protein interaction (PPI), Least Absolute Shrinkage and Selection Operator (LASSO), and Support Vector Machine Recursive Feature Elimination (SVM-RFE) methodologies was employed to screen hub genes associated with CTEPH, which were further verified by dataset GSE188938, quantitative real-time polymerase chain reaction (qRT-PCR) and Western blot. CIBERSORT was utilized to evaluate the infiltration of immune cells and the relationship between infiltration-related immune cells and ARGs. Finally, targeted drug analysis and molecular docking were used to predict drugs targeting Anoikis process to treat CTEPH. Thirty-two differentially expressed genes related to Anoikis and CTEPH were screened through WGCNA analysis. Then, the key ARGs *FASN*, *PLAUR*, *BCL2L1*, *HMOX1* and *RHOB* were screened by PPI, Lasso and SVM-RFE machine learning. Validation through dataset GSE188938, qRT-PCR, and Western blot analyses confirmed *HMOX1* and *PLAUR* as powerful and promising biomarkers in CTEPH. In addition, CIBERSORT immunoinfiltration revealed that Mast_cells_activated and Neutrophils were involved in the pathological regulation of CTEPH. Correlation analysis indicated that *HMOX1* was positively correlated with Neutrophils, while *PLAUR* was negatively correlated with Mast_cells_activated. Finally we used targeted drug analysis and molecular docking to identify that STANNSOPORFIN as a potential drug targeting *HMOX1* for the treatment of CTEPH. *HMOX1* and *PLAUR* emerge as potential biomarkers for CTEPH and may influence the development of CTEPH by regulating Anoikis. Mast_cells_activated and Neutrophils may be involved in Anoikis resistance in CTEPH patients, presenting novel insights into CTEPH therapeutic targets. STANNSOPORFIN is a potential agents targeting Anoikis process therapy for CTEPH.

## Introduction

Chronic thromboembolic pulmonary hypertension (CTEPH) is a non-neoplastic chronic condition characterized by the persistent obstruction of pulmonary arteries by blood clot, resulting in altered blood flow and pulmonary microvascular remodeling. CTEPH belongs to the fourth category of pulmonary hypertension, and severe CTEPH can precipitate right heart failure and increase mortality rates^1^. The diagnostic criteria for CTEPH include radiographically confirmed chronic pulmonary thrombosis after three months of standardized anticoagulant therapy, mean pulmonary artery pressure of ≥ 20 mm Hg, and pulmonary artery wedge pressure ≤ 15 mm Hg^2^. Epidemiological studies suggest that the prevalence of CTEPH at approximately 30 per 1,000,000 individuals. Early symptoms of CTEPH are usually not obvious, and patients with severe CTEPH have a significant mortality rate, with a 5-year survival rate of less than 40%^3^. The pathogenic mechanisms of CTEPH remain elusive, with hypercoagulability, fibrinolysis disorders, inflammation, and pulmonary microvascular hyperplasia among the suspected contributing factors^4^. Early diagnosis of CTEPH is challenging due to the absence of specific biomarkers that predict disease severity and clinical outcomes, highlighting the need for further investigation into potential genetic biomarkers that could reveal the underlying etiology and therapeutic targets.

Anoikis, a specialized form of apoptosis triggered by cell detachment from the extracellular matrix, plays a pivotal role in body development, tissue homeostasis, disease pathogenesis, and tumor metastasis. At present, experts posit that Anoikis maintains cellular-environmental balance by inhibiting the reattachment and proliferation of detached cells in inappropriate locations^5^. However, when the Anoikis process is disrupted, detached cells can proliferate uncontrollably, affecting overall bodily function. It has been demonstrated that stimulating the Anoikis process and enhancing stem cell adhesion at the site of injury can treat pulmonary arterial hypertension (group 1 pulmonary hypertension)^6^. Despite similarities in the pathological mechanisms of various pulmonary hypertension types, the role of Anoikis in CTEPH (group 4 pulmonary hypertension) has not been explored. Therefore, this study aims to investigate the potential weakening of the Anoikis process in CTEPH, which could have significant implications for early and precise diagnosis, as well as the identification of new therapeutic targets.

In this study, we endeavored to identify Anoikis-related prognostic biomarkers in CTEPH using microarray sequencing data from GEO database and machine learning techniques. We also further examined the biological effects and immune infiltration associated with these biomarkers. And Anoikis-related diagnostic markers were validated by dataset GSE188938, qRT-PCR and Western blot. Finally, the drugs targeting Anoikis process were predicted through molecular docking. This work introduces innovative perspectives and therapeutic target for early clinical diagnosis of CTEPH.

## Materials and methods

### Microarray data and differential expression analysis

**Fig. 1:**
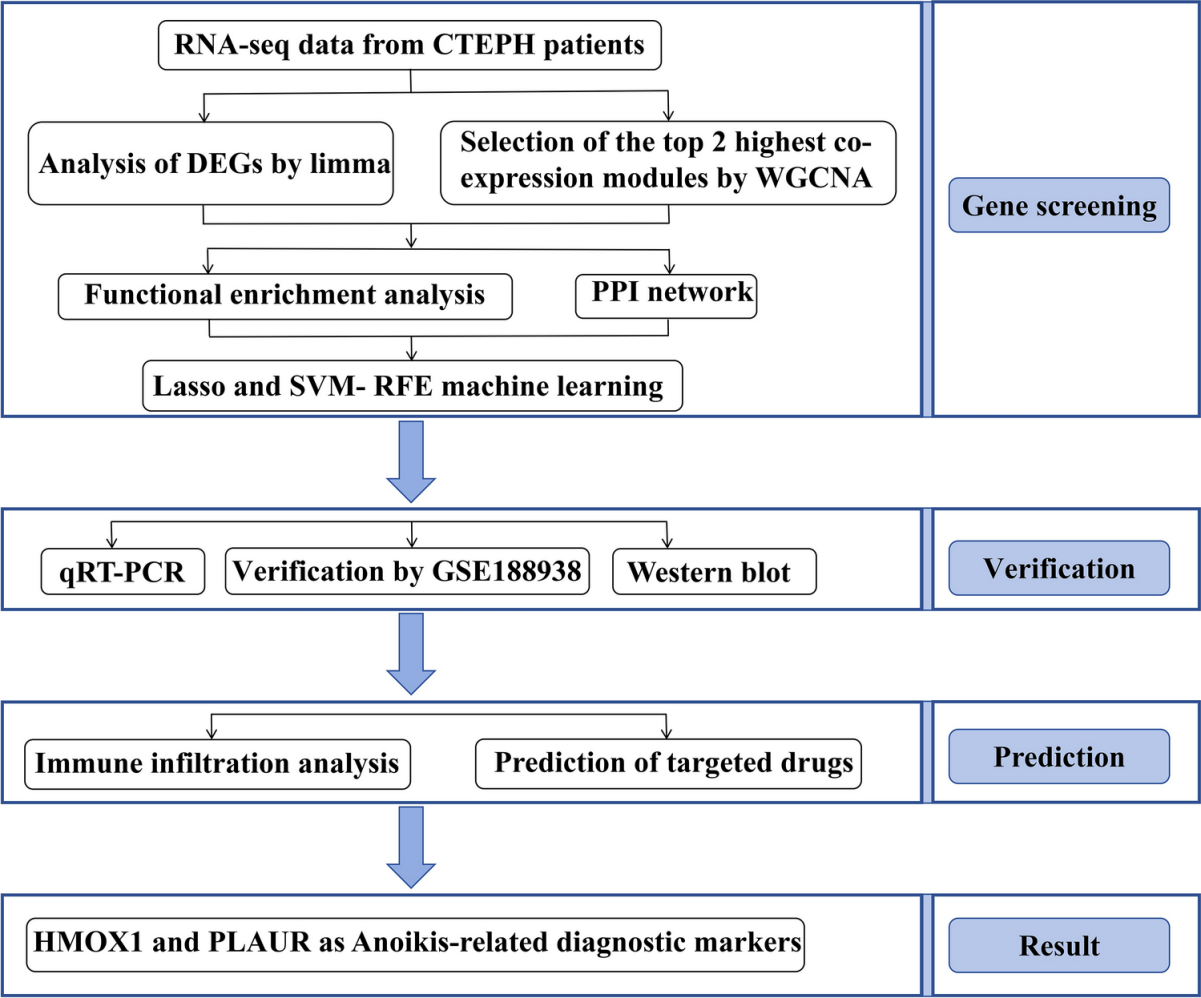
Flowchart of the multistep screening and verification strategy on bioinformatics data and in vitro experiment. *CTEPH* chronic thromboembolic pulmonary hypertension, *DEGs* differentially expressed genes, *WGCNA* weighted gene co-expression network analysis, *PPI network* protein–protein interaction network, *LASSO* least absolute shrinkage and selection operator, *SVM-RFE* support vector machine recursive feature elimination, *qRT-PCR* quantitative reverse transcription polymerase chain reaction.

The study flow chart is depicted in Fig. 1. Using the R software’s “GEOquery” tool (version 4.2.2), the CTEPH dataset GSE130391 was acquired from the GEO (https://www.ncbi.nlm.nih.gov/geo/)^7,8^. The dataset contained the CTEPH group (n = 14) and the Control group (n = 4). Inclusion criteria for CTEPH patients who met the study requirements were chronic pulmonary thrombosis demonstrated on imaging after 3 months of standardized anticoagulation therapy, with mean pulmonary artery pressure ≥ 20 mm Hg, pulmonary artery wedge pressure ≤ 15 mm Hg, and 6 Minute Walk Test < 332 m. The control group had no underlying other diseases. We performed differentiation analysis on the expression of all genes in Control and CTEPH samples using the “limma” package in R software to find differentially expressed genes (DEGs) in CTEPH. The genes with p < 0.05 and |log2FC| > 1 were considered significant. R codes were showed in Supplementary Material 1 and the data of R codes were showed in Supplementary Material 2.

### Weighted gene co-expression network analysis (WGCNA) and module gene selection

Based on the differential genes mentioned above, we screened for additional important genes using the “WGCNA” software. WGCNA is a methodical biological approach to describing gene association patterns across samples. It can be used to identify highly variable gene sets as well as candidate biomarker genes or therapeutic targets based on gene set interconnectedness and the relationship between gene sets and phenotypes^9^. To begin, the outlier genes and samples were removed using the goodSamplesGenes method of the R software package WGCNA. In addition, weighted expression correlations were used to construct gene co-expression networks. Third, a hierarchical clustering analysis based on weighted correlation was performed, and the segmentation and clustering results were performed using the topological overlap matrix (TOM) = 30 to obtain different gene modules, which were represented by branches and different colors of clustering trees. Fourth, the relationship between gene modules and phenotype was calculated, and trait-related modules were identified. Finally, in order to see the network of interactions between different models at the system level, the relationships between the models are finally examined.

### Identification of differentially expressed Anoikis-related genes

A total of 434 Anoikis-related genes were obtained from GeneCards, and the selection criterion was set as correlation score > 0.4 (Supplementary Material 3)^10^. To investigate the connection between Anoikis and CTEPH, the differential genes and Anoikis-related genes were intersected. These differentially expressed Anoikis-related genes (ARGs) are thought to be key regulators of CTEPH by Anoikis.

### Functional enrichment analysis

Gene Ontology (GO) analysis is a widely used technique in large-scale functional enrichment research projects. Gene function is classified as three categories: Biological Process (BP), Molecular Function (MF) and Cellular Component (CC). KEGG (Kyoto Encyclopedia of Genes and Genomes) is a popular database for predicting biological pathways. Immunologic-related enrichment assays for previously reported target genes were combined to generate the Immunologic Signatures database, which is used for immunomarker enrichment analysis. Reactome is a database of articles on various human responses and biological pathways. The Database for Annotation, Visualization, and Integrated Discovery (DAVID) tools (https://david.ncifcrf.gov/) were used to perform GO and KEGG. p < 0.05 was regarded as statistically significant^11^. Immunologic Signatures and Reactome enrichment associated with ARGs were examined using Metascape (http://metascape.org/). p < 0.01 and gene counts ≥ 5 were considered statistically significant^12^.

### Protein–protein interaction network construction and hub genes identification

The Search Tool for the Retrieval of Interacting Genes (STRING) Database (http://string-db.org/) is a database that can be used to analyze the relationships between interacting genes. For the purpose of assessing possible PPI relationships, the previously detected ARGs are added to the STRING database. The genes with PPI comprehensive score > 0.4 were identified as the key genes^13^.

### Machine learning

Least absolute shrinkage and selection operator (Lasso) is an L1-regularized linear regression machine learning method and commonly utilized for feature selection or as a binary classifier^14^. To achieve the goals of sparsification and feature selection, the weights of some learned features was set to 0. The LASSO method identifies essential features from large-scale data and selects ARGs genes that exhibit significant differential expression between CTEPH and control groups. This is done by using p-value selection and selecting more predictive and less linearly dependent genes^15^. We integrated survival status and gene expression data from the GSE130391 dataset using the ‘glmnet’ package in R software and performed regression analysis with the LASSO-Cox method. To optimize the model, the tenfold cross validation was used to find the best model. SVM-RFE (support vector machine-recursive feature elimination) is a sequential backward selection algorithm based on SVM’s maximum interval principle. This algorithm ranks features by their scores in the model training phase and eliminates those with the lowest scores, repeating the training process with the remaining features in subsequent iterations until the desired number of features is reachedd^16^. In summary, the SVM-RFE algorithm can eliminate noise or redundant features, thereby reduce data dimensionality and enhance model accuracy to identify the most important differentially expressed genes of CTEPH. Finally, we intersected the diagnostic markers identified by both Lasso and SVM-RFE to determine the most robust genetic diagnostic markers for CTEPH.

### Verification of optimal potential biomarkers for CTEPH in dataset GSE188938

The gene expression profile of CTEPH-related dataset GSE188938 was obtained using R software (Control, n = 5; CTEPH, n = 7). Five potential biomarkers (*FASN*, *PLAUR*, *BCL2L1*, *HMOX1* and *RHOB*) were compared between the Control group and the CTEPH group using SPSS software. The expression data were analyzed by T test to determine differences between the Control group and the CTEPH group. p < 0.05 is considered a statistical difference.

### Quantitative PCR analysis

To further verify the prediction results above, quantitative reverse transcription polymerase chain reaction (qRT-PCR) was used to detect the expression of diagnostic markers *HMOX1* and *PLAUR* in the serum of CTEPH patients. Total RNA was extracted from each sample using TRIzol reagent, followed by reverse transcription into cDNA using a cDNA reverse transcription kit. The cDNA was then amplified with the SYBR Green PCR kit (Qiagen, Germany). GAPDH was used as a standardized endogenous control and p < 0.05 was statistically significant. The relative expression of mRNA was calculated using the ∆CT (Ct mRNA-Ct GAPDH) method, while the 2^−∆Ct^ method was applied to determine the relative quantification values for mRNA. Primer sequences included GAPDH forward, CTCTCTCTACACAGCGACA, GAPDH reverse, GCT GTAGCCAAATTCGTTGTCAT; *PLAUR* forward, GGTGACGCCTTCAGCATGA, *PLAUR* reverse, CCCACTGCGGTACTGGACAT; *HMOX1* forward, CCTCCCTGTACCACATCTATGT, *HMOX1* reverse, GCTCTTCTGGGAAGTAGACAG. The study was approved by the institutional review board and the ethics committee of Henan Provincial People’s Hospital (Ethical Review No.: 2020-158). Informed Consent was obtained from patients and volunteers for the study. And we confirmed that the authors strictly complied with the Declaration of Helsinki. The PCR expression data were statistically analyzed by T test to determine differences between the Control group and the CTEPH group.

### Western blot

Diagnostic markers *HMOX1* and *PLAUR* were further verified by Western blot between CTEPH patients and healthy person. Equal amounts of protein were added to the pre-cooled RIPA lysate (100:1:1, RIPA: PMSF: phosphatase inhibitor lysate before use) for cleavage before processing. The total protein concentration in the lysate was measured using the BCA assay. A total of 30 μg of protein was resolved by 10% SDS-PAGE and then transferred onto nitrocellulose membranes. The membranes were blocked with 5% skim milk powder and incubated overnight at 4 °C with primary antibodies specific for *HMOX1* (diluted 1:2000) and *PLAUR* (diluted 1:1000). On the following day, the membranes were incubated with an IgG-HRP secondary antibody (diluted 1:5000) for 2 h at room temperature. The immunoreactive bands were visualized using an ECL chemiluminescent substrate. The grayscale values of the bands, quantified using ImageJ software, correspond to the relative protein expression levels. The Western blot data were statistically analyzed using a T-test to assess the significance of differences in protein expression between the control group and the CTEPH group.

### Immune infiltration analysis

CIBERSORT (https://cibersort.stanford.edu/) is a computational method used to deconvolute the gene expression profiles of complex tissues into the constituent proportions of 22 distinct immune cell types^17^. The SangerBox tool was used to compare the percentages of 22 immune cell infiltrates between CTEPH and Control group. We then generated a correlation heatmap and box plot to visualize and analyze the significant differences in immune cell composition between the CTEPH and Control groups. The gene expression data for each of the 22 immune cell types were statistically evaluated using a T-test to identify any significant differences in their relative abundance between the Control and CTEPH groups.

### Correlation analysis between ARGs and infiltration-related immune cells

SPSS software was used to analyze the Spearman rank correlation between ARGs identified (*HMOX1* and *PLAUR*) and the level of related immune cells. p < 0.05 was considered statistically significant. Additionally, the ‘ggplot2’ package in R software was utilized to visually represent the correlations.

### Prediction of ARGs-targeted drugs

Drug Gene Interaction Database (DGIdb) (https://www.dgidb.org/) was used to predict drugs that may target the identified diagnostic markers^18^. Subsequently, a diagnostic markers-targeted drug network was constructed using Cytoscape software, with the drug exhibiting the highest interaction score being deemed the most important. To further elucidate the interactions between these drugs and diagnostic markers, molecular docking analysis was conducted. The 3D structures of diagnostic markers and drug were obtained from the RCSB PDB database (https://www.rcsb.org/) and the pubchem database (https://pubchem.ncbi.nlm.nih.gov/), respectively. The diagnostic markers protein were dehydrated with PyMOL, hydrogenated, and the charge calculated using AutoDock software. Finally, AutoDockVina was used for docking in order to determine the best conformation and the length of the hydrogen bond^19^.

### Statistical analysis

All the data were showed as the mean ± standard deviation. Data between two or more groups that fit the normal distribution were compared using the T test or one-way ANOVA. The Wilcoxon rank sum test was employed to examine non-normally distributed variables. Every statistical test was two-sided. For all statistical tests, p < 0.05 was considered as statistically significant.

## Result

### Identification of differentially expressed genes in CTEPH

**Fig. 2:**
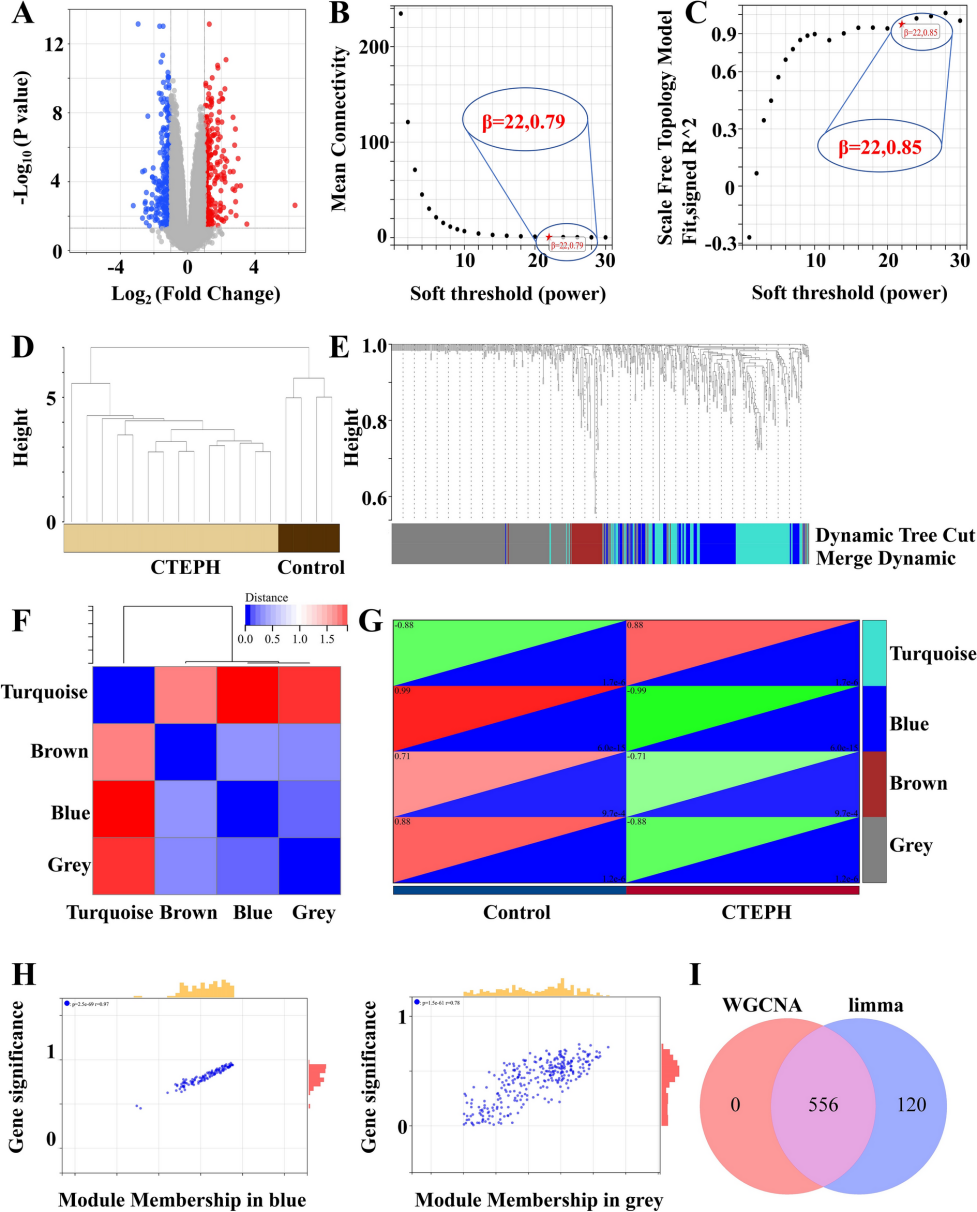
Identification of differentially expressed genes in CTEPH by limma and WGCNA packages. (**A**) PCA diagram of the DEGs expression. Red represents gene up-regulation and blue represents gene down-regulation. (**B**) Scale independence diagram. (**C**) Average connectivity graph. The soft threshold is 22. (**D**) Hierarchical clustering tree of MRNA expression patterns of 18 samples. Sample aggregation, no outlier samples. (**E**) Correlation diagram between modules. (**F**) Dendrogram of all differentially expressed genes clustered based on different similarity measures. Turquoise, gray, blue and brown are the four modules with the highest degree of clustering. (**G**) Heat map of the characteristic genes of the module. (**H**) Scatterplot of correlation between modules and clinical traits. Gray (r = 0.78, p = 1.5e−61), blue (r = 0.63, p = 2.7e−17). (**I**) Intersection gene map of limma package and WGCNA package.

The volcano map demonstrated significant differences in gene expression between CTEPH patients and healthy individuals. Compared with the control group, we extracted 676 DEGs from the GSE130391 database using R software, of which 341 were down-regulated and 345 were up-regulated in the CTEPH (Fig. 2A).

### Co-expression network of DEGs in CTEPH

We first evaluated the reliability of the network, and found no outlier samples for removal (Fig. 2D). Using the “WGCNA” package, the co-expression network was constructed based on 676 DEGs genes with a soft threshold of 22 (Fig. 2B,C). Four modules were identified by average hierarchical clustering and dynamic tree pruning (Fig. 2E,F). A heat map of the module-feature relationship was used to assess the correlation between the DEG and each module (Fig. 2G). The correlation between blue module and DEGs was the highest (r = 0.97, p = 2.5e−69, Spearman rank correlation coefficient). We found gray (r = 0.78, p = 1.5e−61), blue (r = 0.63, p = 2.7e−17) and brown (r = 0.38, p = 7.9e−3) (Fig. 2H). Consequently, we focused on the blue and gray modules for further analysis, encompassing a total of 556 DEGs (Fig. 2I).

### Identification of differentially expressed Anoikis-related genes

**Fig. 3:**
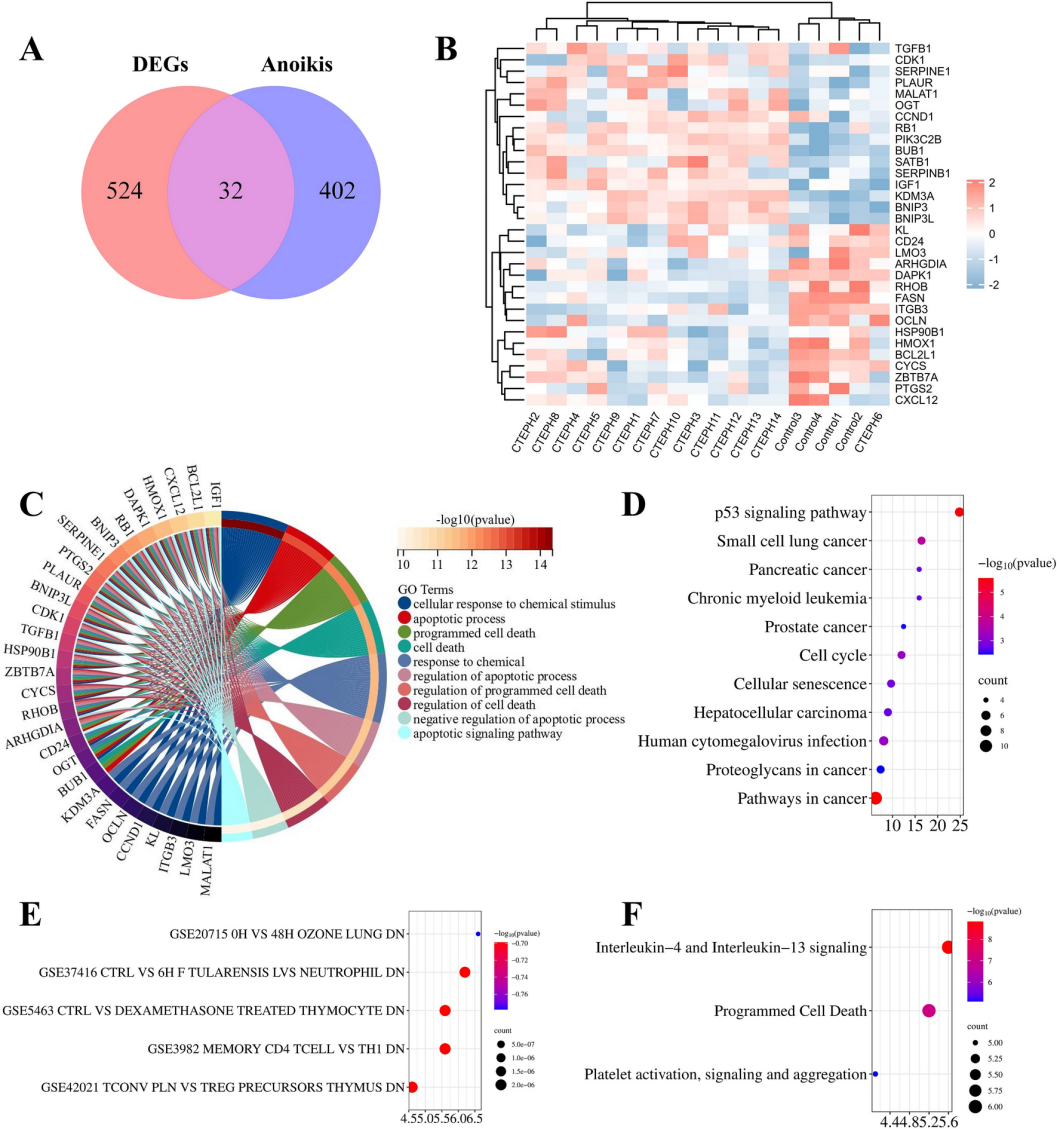
Identification of differentially expressed Anoikis-related genes and functional enrichment analysis. (**A**) Venn diagram of DEGs and Anoikis to get 32 Anoikis-related genes. (**B**) The clustering heatmap of the expression pattern ofdifferentially expressed Anoikis-related genes. The abscissa represents samples, and the abscissa represents genes. Red for gene up-regulation and blue for gene down-regulation. (**C**) GO enrichment analysis. (**D**) KEGG enrichment analysis. (**E**) Immunologic Signatures enrichment analysis. (**F**) Reactome enrichment analysis. The Y axis represents the pathway name. The X-axis represents the ratio of the number of genes enriched to the target pathway to the total number of target genes. The color represents the adjusted p-values, the redder the color or the smaller the p value, the more significant the enrichment. The larger the bubble, the more genes present in each pathway.

There were 434 Anoikis-related genes with correlation score > 0.4 obtained from GeneCards (Supplementary Material 3). From these, we identified 32 ARGs from Anoikis and DEGs (Supplementary Material 4) (Fig. 3A). The clustering heatmap visually depicted the expression of 32 differentially expressed Anoikis-related genes between CTEPH group and Control group (Fig. 3B).

### Functional enrichment analysis

GO analysis results showed that differentially expressed Anoikis-related genes were predominantly related to cellular response to chemical stimulus apoptotic process, programmed cell death cell death, regulation of apoptotic process, regulation of programmed cell death regulation of cell death, negative regulation of apoptotic process and apoptotic signaling pathway (Fig. 3C). KEGG enriched in Pathways in cancer p53 signaling pathway, Cell cycle and Cellular senescence (Fig. 3D). The DE-ARGs exhibited notable enrichment in many immune- related signatures, which indicated that DE-FRGs may play a role in the pathogenesis of CTEPH by participating in the regulation of immune cells, cytokines and inflammation (Fig. 3E). Reactome enrichment enriched in Interleukin-4 and Interleukin-13 signaling, Programmed Cell Death Platelet activation and signaling and aggregation (Fig. 3F).

### PPI network and machine learning

**Fig. 4:**
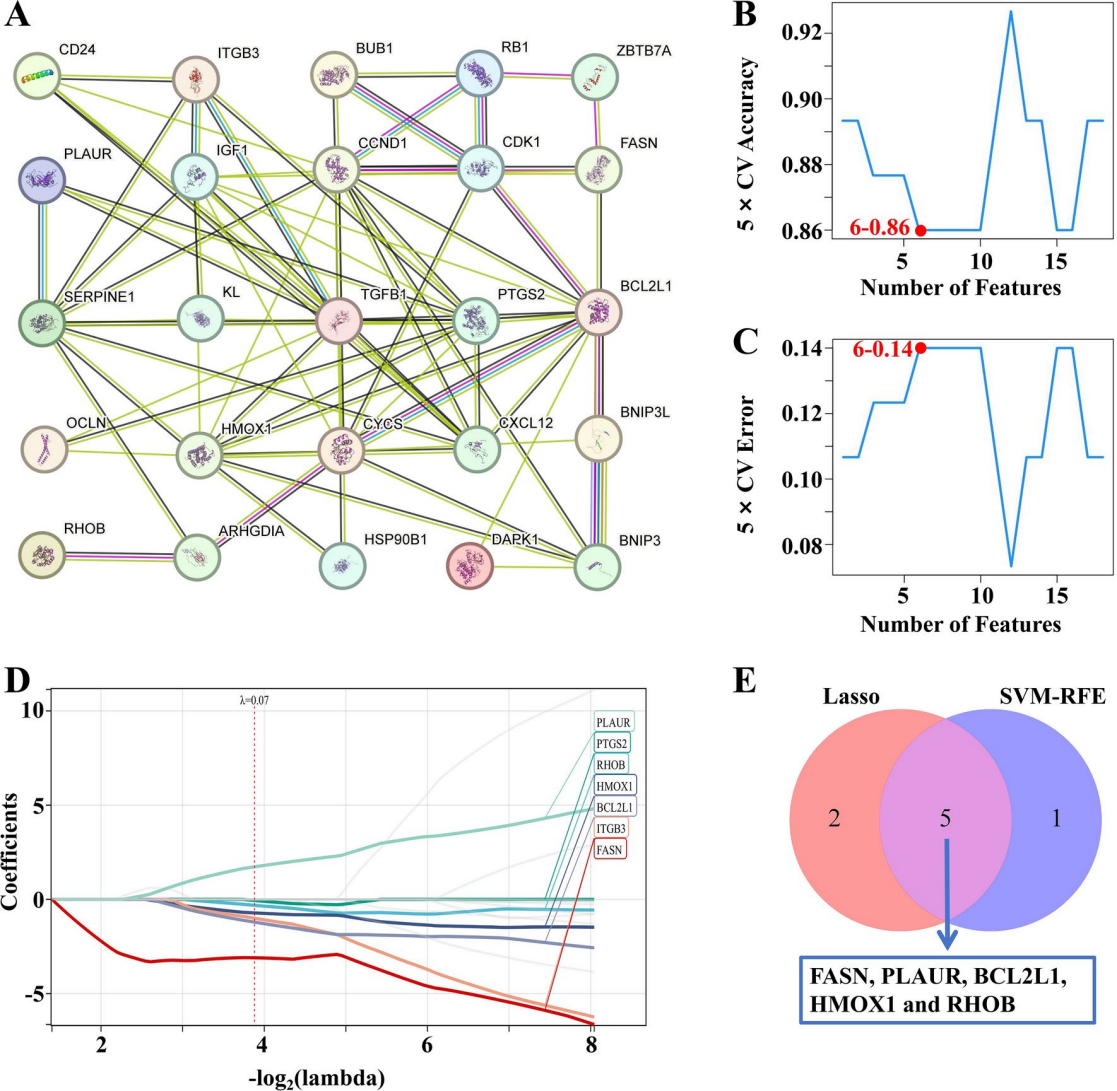
PPI network and machine learning. (**A**) PPI network. The number of lines between the two proteins represents the strength of the interaction. (**B**) 5 × CV Accuracy of SVM-RFE machine learning. (maximal accuracy = 0.86). The X axis is the number of features and the Y axis represents the accuracy of the curve change after 5 times cross-validation. 6–0.86 means that the accuracy rate of 6 features is 0.86. The closer the accuracy is to 1, the higher the accuracy. (**C**) 5 × CV Error of SVM-RFE machine learning. (minimal RMSE = 0.14). The X axis represents the number of features, and the Y axis represents the error rate of curve changes after 5 times cross-validation. 6–0.14 indicates that the error rate of 6 features is 0.14. The closer the accuracy is to 0, the lower the error rate. (**D**) Lasso machine learning. Lasso machine learning obtained 7 genes that contributed more to the model prediction through regression analysis and cross-validation. (**E**) Venn diagram of Lasso and SVM-RFE machine learning. *PPI network* protein–protein interaction network, *LASSO* least absolute shrinkage and selection operator, *SVM-RFE* support vector machine recursive feature elimination.

The protein interactions between differentially expressed Anoikis-related genes were predicted using the STRING tool, resulting in a network comprising 24 nodes and 66 edges in the PPI network. The 24 differentially expressed Anoikis-related genes were identified as key genes (Fig. 4A). Penalty parameters were tuned by tenfold cross validation, and 6 genes were identified as diagnostic markers from differentially expressed Anoikis-related genes using SVM-RFE algorithm (Fig. 4B,C). Subsequently, LASSO machine learning was utilized to screen out 7 genes from differentially expressed Anoikis-related genes as diagnostic markers of CTEPH (Fig. 4D). By intersecting the diagnostic markers obtained from the two algorithms and finally five diagnostic phase markers (*FASN*, *PLAUR*, *BCL2L1*, *HMOX1* and *RHOB*) were obtained (Fig. 4E). These markers collectively represent promising candidates for the diagnosis of CTEPH, highlighting their potential utility in clinical practice.

### Verification of optimal potential biomarkers for CTEPH in dataset GSE188938

**Fig. 5:**
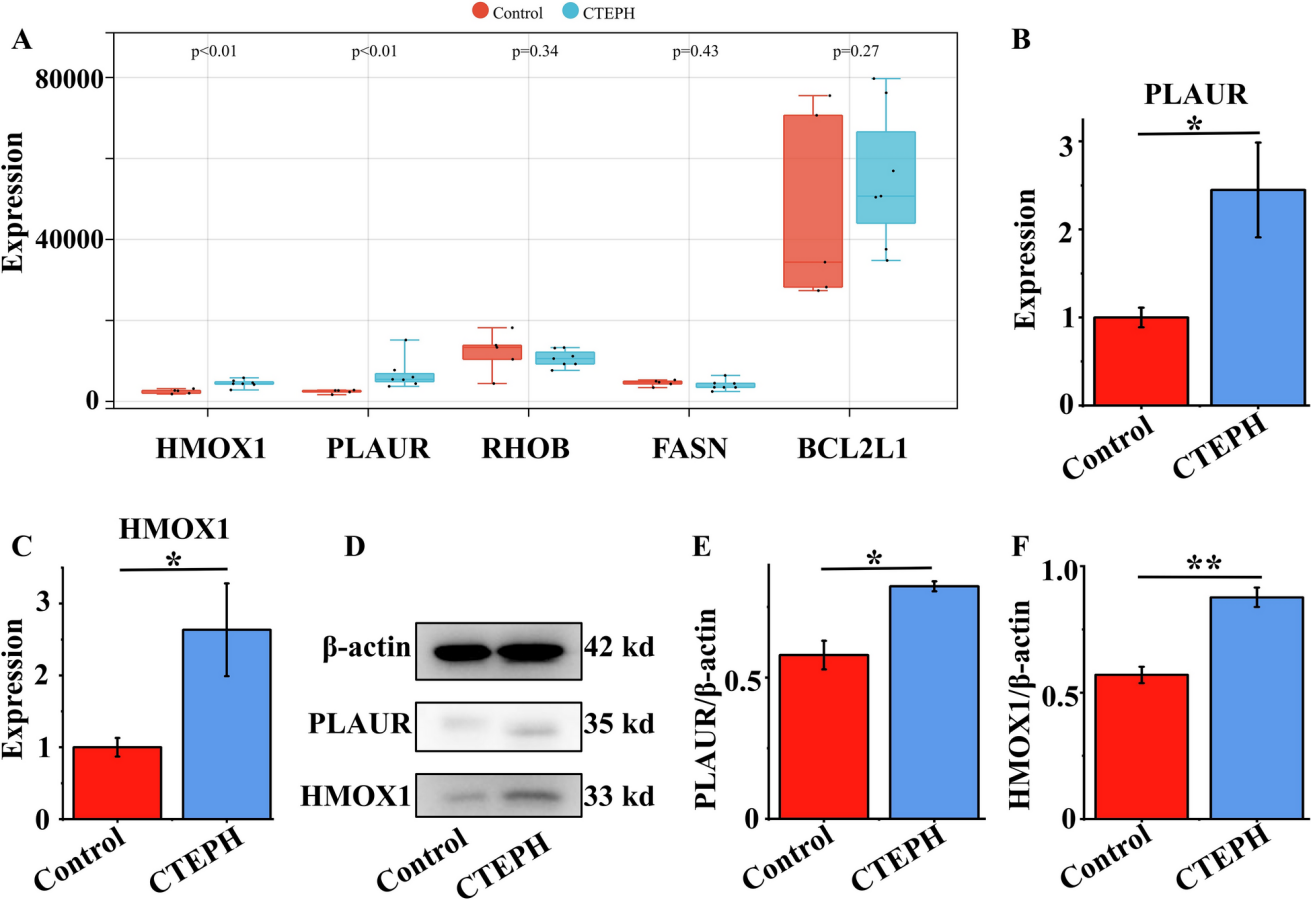
Verification of *PLAUR* and *HMOX1* by dataset GSE188938, qRT-PCR and Western blot. (**A**) Expression of *HMOX1*, *PLAUR*, *RHOB*, *FASN* and *BCL2L1* in CTEPH and Control group of dataset GSE188938. (**B,C**) Validation of the expressions of potential diagnostic markers via qRT-PCR, n = 5. (**D–F**) Validation of the expressions of potential diagnostic markers via Western blot, n = 3. *p < 0.05; **p < 0.01. *qRT-PCR* quantitative reverse transcription polymerase chain reaction.

To make our study more convincing, we used R software to obtain gene expression profiles of CTEPH-related dataset GSE188938 (Control, n = 5; CTEPH, n = 7). Upon analysis, we observed that only *HMOX1* (p = 0.0051) and *PLAUR* (p = 0.0025) exhibited statistically significant differences, whereas *FASN*, *BCL2L1*, and *RHOB* did not (Fig. 5A). This suggests that *PLAUR* and *HMOX1* may be the most important diagnostic markers. Specific data were shown in Supplementary Material 5.

### Quantitative PCR analysis and western blot

Table 1 presents the medical information of both CTEPH patients and healthy volunteers. It’s noteworthy that none of the healthy volunteers had any underlying diseases. The CTEPH patients included in the study were all confirmed cases with 6WMT < 332 m, mPAP > 20 mmHg and PAWP ≤ 15 mm Hg. qRT-PCR was adopted to detect the expression levels of *PLAUR* and *HMOX1* in the peripheral blood samples from CTEPH and Control groups. The peripheral blood expression levels of *PLAUR* (p = 0.02985) and *HMOX1* (p = 0.0377) were significantly higher in CTEPH patients than Control group (Fig. 5B,C). To further bolster the credibility of these findings, Western blot was used to further prove that *PLAUR* and *HMOX1* are two important diagnostic markers. And we also found that the expression levels of *PLAUR* (p = 0.01032) and *HMOX1* (p = 0.00361) were significantly higher in CTEPH patients than Control group (Fig. 7D–F). Remarkably, the qRT-PCR and Western blot results corroborated the predicted results of bioinformatics analysis. The raw data of PCR and WB were presented in Supplementary Material 6 and Supplementary Material 7 respectively.

**Table 1:** The clinical information of CTEPH and control group.

Group	Gender	Age	6WMT (m)	mPAP (mmHg)	PVR (Wood units)	PAWP (mmHg)	CI (min·m^2^)
CTEPH	Female	66	316.1	60.4	11.2	7.3	2.3
CTEPH	Female	59	308.8	56.7	13.8	11.7	1.9
CTEPH	Male	69	240.6	59	11.7	12.8	2.4
CTEPH	Male	62	317.3	64.1	9.2	10.4	2.2
CTEPH	Male	57	250.7	61.1	12.4	8.8	2.1
Control	Male	57	–	–	–	–	–
Control	Female	74	–	–	–	–	–
Control	Female	45	–	–	–	–	–
Control	Male	68	–	–	–	–	–
Control	Male	55	–	–	–	–	–

*6MWT* 6 Minute Walk Test, *mPAP* mean pulmonary arterial pressure, *PVR* pulmonary vascular resistance, *PAWP* pulmonary artery wedge pressure, *CI* cardiac Index.

### Immune infiltration analysis

**Fig. 6:**
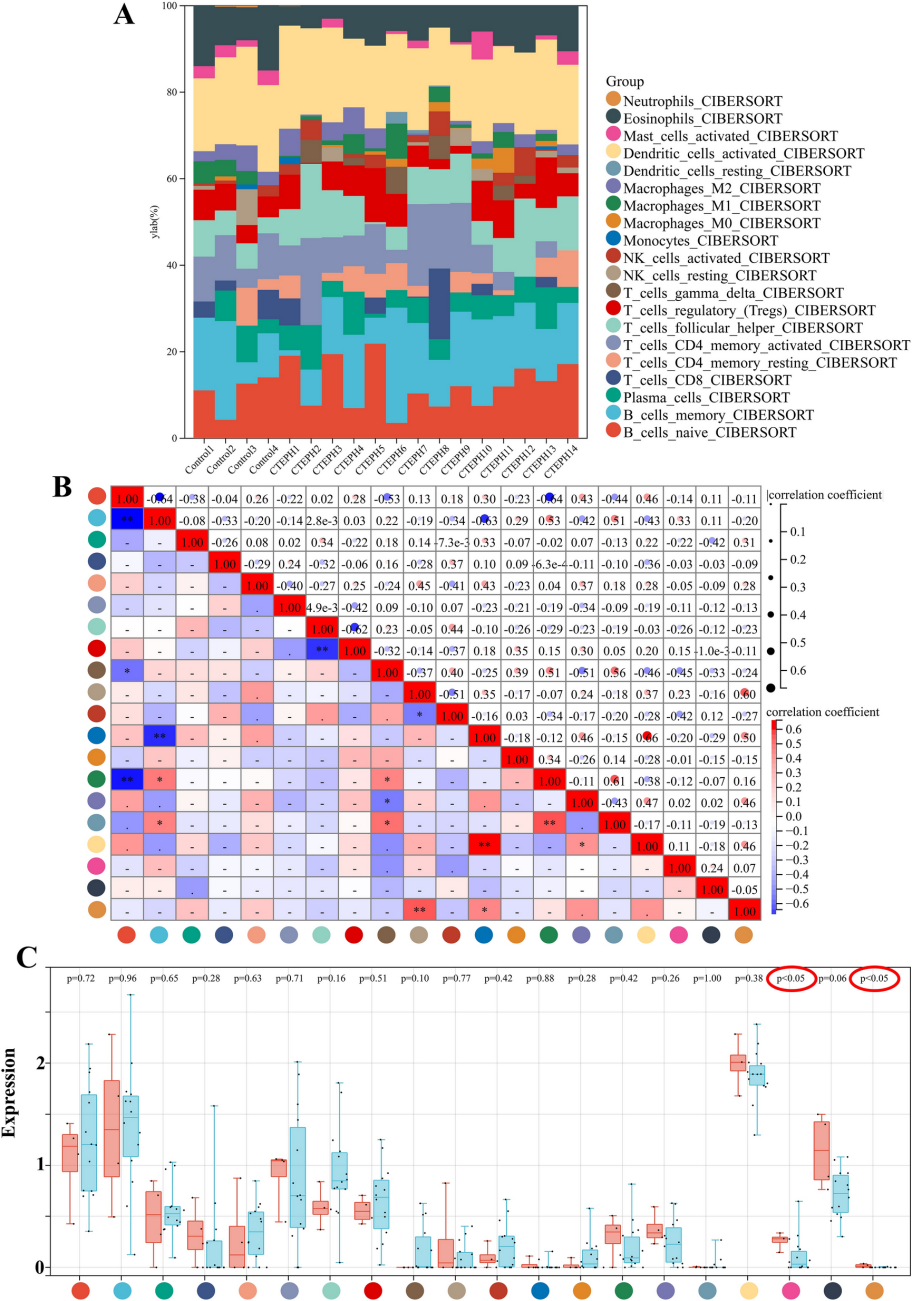
Immune infiltration analysis. (**A**) The proportions of the 22 immune cells in each sample. The X axis represents each sample, and the Y axis represents the proportion of different immune cells. The 22 colors represent 22 types of immune cells. (**B**) The correlation heat map of 22 immune cells. (**C**) Box-type plot of immune cell infiltration differences.

Figure 6A displays the proportions of the 22 immune cells in each sample. The correlation heat map of 22 immune cells revealed a positive correlation between Mast_cells_activated and Neutrophils (Fig. 6B). Additionally, we found that Mast_cells_activated and Neutrophils were statistically significant in box-type plot. The expression of Mast_cells_activated and Neutrophils decreased compared with normal control samples. Such results indicate a potential involvement of decreased Mast_cells_activated and Neutrophils in the pathogenic process of CTEPH (Fig. 6C).

### Correlation analysis between diagnostic markers and infiltration-related immune cells

**Fig. 7:**
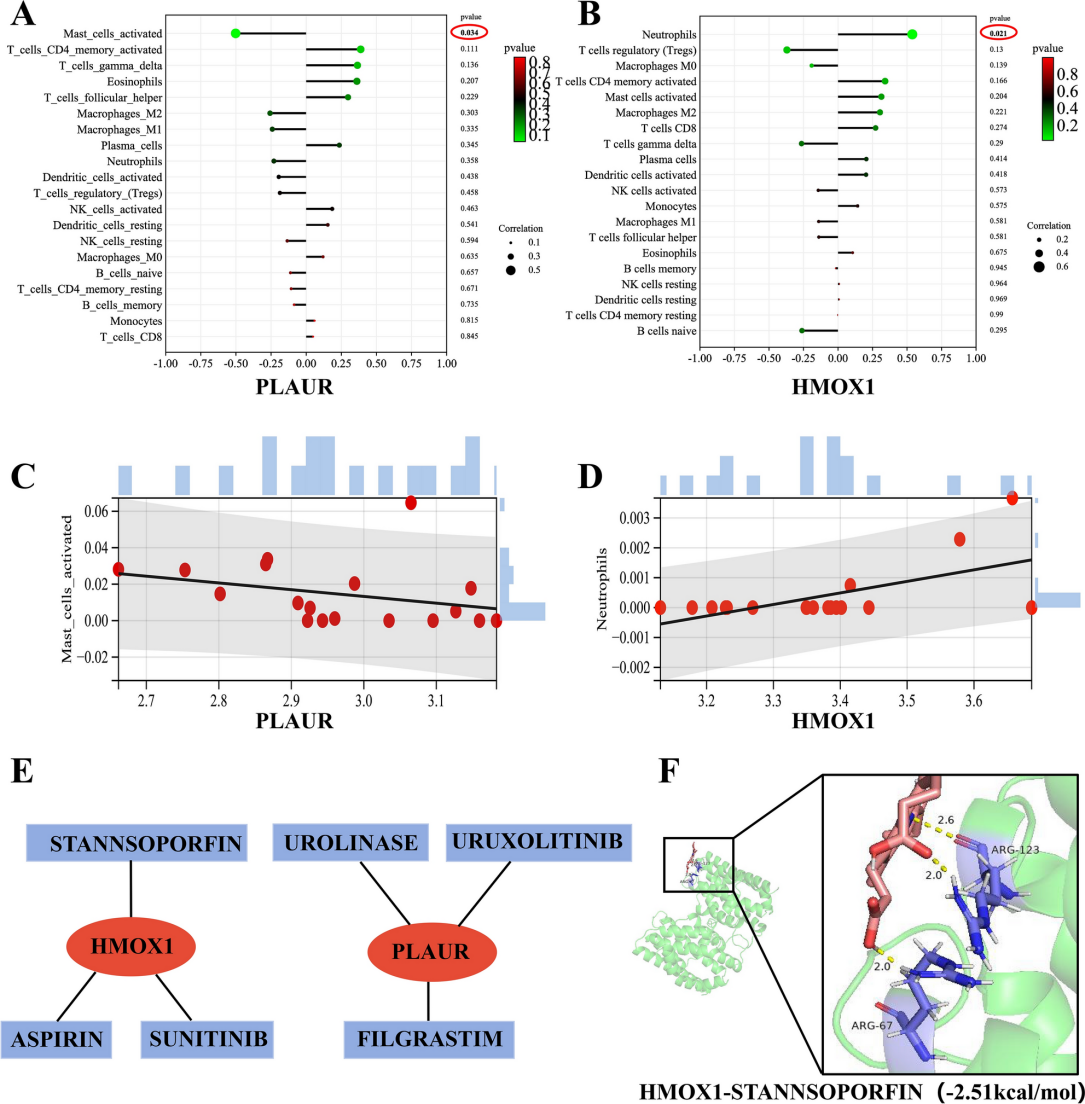
Correlation analysis between diagnostic markers and infiltration-related immune cells and construction of diagnostic markers-targeted drugs network. (**A**) Correlation analysis between *PLAUR* and infiltration-related immune cells. (**B**) Correlation analysis between *HMOX1* and infiltration-related immune cells. (**C**) Scatter diagram indicating the correlation between *PLAUR* expression and Mast cells activated. The expression of *PLAUR* was negatively correlated with Mast_cells_activated (r =  − 0.502, p = 0.034). (**D**) Scatter diagram indicating the correlation between *HMOX1* expression and Neutrophils. The expression of *HMOX1* was positively correlated with Neutrophils (r = 0.538, p = 0.021). (**E**) *PLAUR* and *HMOX1*-targeted drugs network. Red represented diagnostic markers and light blue represented the drugs. (**F**) Molecular docking of diagnostic markers- targeted drugs. The binding energies of *HMOX1*-STANNSOPORFIN is − 2.51 kcal/mol.

Correlation analysis revealed that *PLAUR* was negatively correlated with Mast_cells_activated (r =  − 0.502, p = 0.034) (Fig. 7A,C). *HMOX1* was positively correlated with Neutrophils (r = 0.538, p = 0.021) (Fig. 7B,D).

### Construction of diagnostic markers-targeted drugs network

The Cytoscape software visualization results of diagnostic markers-targeted drugs were presented in Fig. 7E. We identified six diagnostic markers-targeted drugs, comprising three associated with *PLAUR*, and three of *HMOX1*. Drugs with an interaction score > 2 were deemed to be well-matched with the diagnostic markers, and molecular docking was performed accordingly. Among these, only *HMOX1-*targeted drug (STANNSOPORFIN) possessed the 3D structure and its binding energy < 0. Specifically, the binding energies of *HMOX1*-STANNSOPORFIN was − 2.51 kcal/mol (Fig. 7F). These findings suggest that STANNSOPORFIN is likely to have a therapeutic effect on CTEPH.

## Discussion

Despite considerable advancements in the management of CTEPH over recent decades, pharmacological treatments and Pulmonary Endarterectomy (PEA) have not uniformly achieved the desired therapeutic outcomes, leaving many critically ill patients at risk of relapse and mortality. As sequencing technology continues to evolve, there is a growing anticipation among researchers to delve into the intricate biological effects of mRNA in Chronic Thromboembolic Pulmonary Hypertension (CTEPH). This exploration is driven by the potential to identify early indicators of CTEPH, enabling preemptive medical interventions that could significantly alter the course of the disease. In the realm of pulmonary arterial hypertension (Group 1 pulmonary hypertension), a diminished Anoikis process has been implicated in disease progression. However, scant research has examined the possibility of a similar weakening of the Anoikis process in CTEPH patients, which has profound implications for patients with CTEPH. The pathogenesis of CTEPH is hypothesized to involve a complex interplay of factors, including blood hypercoagulation, fibrinolysis, inflammation, aberrant migration of pulmonary vascular endothelial and smooth muscle cells and so on^20,21^. Anoikis maintains a balance between cells and their surroundings by preventing shed cells from reattaching to new substrates and growing in the wrong places^22^. Therefore, we hypothesized that the absence of Anoikis process or the weakening of Anoikis executive ability may lead to abnormal migration and proliferation of shed pulmonary vascular endothelial cells or smooth muscle cells, leading to the narrowing of pulmonary blood vessels, which is conducive to the formation of white and red thrombosis, and ultimately to CTEPH. In this study, we harnessed machine learning and other bioinformatics methods to preliminarily establish the involvement of Anoikis in CTEPH pathogenesis, identified prognostic biomarkers related to Anoikis, and verified the bioinformatics findings through in vitro experiments. Finally, employing molecular docking technology, we predicted a drug candidate that targets the Anoikis process, offering a novel therapeutic strategy for CTEPH. This research introduces innovative perspectives and potential therapeutic targets for the early clinical diagnosis of CTEPH, with broad implications for foundational research, clinical diagnostics, and treatment strategies in the field.

In this study, we identified 556 differentially expressed genes between the CTEPH group and the control group using the limma package and WGCNA analysis, indicating significant genetic alterations in CTEPH patients. Through analysis of these differential genes, 32 Anoikis-related genes were identified in the differential genes, suggesting the presence of Anoikis deletion or hypofunction processes in CTEPH patients. GO analysis is primarily involved in the regulation of cell death process. In the results of KEGG enrichment analysis, there were significantly enriched in p53 signaling pathway, cell cycle, and cell senescence. Reactome enrichment analysis was mainly associated with cell death, platelet activation, signal transduction and aggregation. Previous studies have proposed that progressive pulmonary vascular remodeling in CTEPH is characterized by abnormal apoptosis of lung smooth muscle cells, leading to pronounced proliferation and long-term progression of pulmonary hypertension^23^. These results suggest that the attenuation of Anoikis process may affect the pathological process of CTEPH, leading to the abnormal process of programmed death of shed lung smooth muscle cells and endothelial cells, resulting in abnormal proliferation and further remodeling and narrowing of pulmonary blood vessels.

Using PPI, LASSO and SVM-RFE machine learning techniques, we identified five Anoikis-related genes (*FASN*, *PLAUR*, *BCL2L1*, *HMOX1* and *RHOB*) that are likely pivotal in the attenuation of Anoikis in CTEPH. Studies have shown that *FASN*-mediated anti-Anoikis promotes the growth and metastasis of osteosarcoma^24^. TCF7L2 enhances Anoikis resistance and metastasis of gastric cancer through transcriptional activation of *PLAUR*^25^. rBMSCs/ITGA5B1 increases human vascular smooth muscle cell development by increasing *HMOX1* expression, hence preventing Anoikis formation^26^. Dexamethasone can influence the expression of *BCL2 L1* in thyroid cancer cells to promote the weakening of Anoikis process^27^. Inhibition the production of* RHOB* protein inactivates the FAK pathway and activates the Anoikis process^28^. Simultaneously, research has revealed that *FASN*, *PLAUR*, *BCL2L1*, *HMOX1* and *RHOB* are involved in the regulation of CTEPH pathological process^29^. The above studies indicate that these major genes are mostly engaged in metabolic regulation, cell replication and other processes closely related to the regulation of cell death. Anoikis is a special programmed apoptosis initiated by cell detachment from extracellular matrix. Metabolic regulation is involved in managing the state of shed cells during the anti-Anoikis process. At the same time, abnormal apoptosis and abnormal replication of cells, the intracellular death process is weakened, and finally the ectopic replication growth can be achieved^30^. The development of CTEPH may be linked to abnormal cell metabolism, abnormal apoptosis of pulmonary artery smooth muscle cells and endothelial cells, and weakened DNA repair function leading to abnormal migration and proliferation, lipid accumulation, and abnormal carbohydrate metabolism^20^. These results suggest that *FASN*, *PLAUR*, *BCL2L1*, *HMOX1* and *RHOB* may participate in the anti-Anoikis process of CTEPH, which further aggravates the condition of patients with CTEPH, which is consistent with the results of our study.

To demonstrate that *FASN*, *PLAUR*, *BCL2L1*, *HMOX1* and *RHOB* are powerful and promising diagnostic biomarkers of CTEPH for Anoikis, we conducted validation experiments utilizing dataset GSE188938, qRT-PCR, and Western blot to assess the expression of Anoikis-related genes in the CTEPH and control groups. And we evaluated that *HMOX1* and *PLAUR* were two of the most important and potential diagnostic biomarkers for CTEPH associated to Anoikis. Lin et al. have suggested that *HMOX1* may be involved in the regulatory processes of CTEPH, including programmed cell death such as Ferroptosis^31^. In addition, *HMOX1*'s role in the regulation of Anoikis is well-documented. Combined with our results, it can be inferred that *HMOX1* may regulate the development of CTEPH through Ferroptosis and Anoikis separately or jointly. Research has indicated that *PLAUR* inhibition leads to a reduction in cancer cell growth, invasion, angiogenesis, and metastasis^32^. Given that the core pathological process of CTEPH is the abnormal growth and invasion of pulmonary artery smooth muscle cells, we speculated that when *PLAUR* is inhibited, the growth, invasion and metastasis of pulmonary artery smooth muscle cells are reduced, which can alleviate the development of CTEPH^33^. And further experimental studies are warranted to elucidate the specific regulatory mechanisms of *HMOX1* and *PLAUR* in CTEPH.

In our study, immune infiltration analysis revealed that Mast_cells_activated and Neutrophils were statistically significant. Correlation analysis showed a positive association between *HMOX1* and Neutrophils, while *PLAUR* was negatively correlated with Mast_cells_activated. Single-cell RNA sequencing analysis has confirmed the involvement of mast cells in the development of CTEPH^34^. Anoikis resistance can be induced by digestive enzymes and trypsin secreted by mast cells^35^. Neutrophil-mediated inflammation has been shown to enhance the TGF-β signaling pathway, contributing to fibrosis and thrombus remodeling, and further aggravating CTEPH^36^. Neutrophils are believed to participate in Anoikis resistance by releasing reactive oxygen species, antimicrobial peptides, and proteases^37^. These findings align with our results, suggesting that both mast cells and neutrophils may play a role in Anoikis resistance in CTEPH patients. The immunocharacteristic enrichment analysis indicated that differentially expressed Anoikis-related genes were also significantly enriched in various immune-related pathways, potentially participating in the pathogenesis of CTEPH and Anoikis by regulating immune cells, cytokines, and inflammation. Overall, above results revealed that *HMOX1* may increase the number of Neutrophils and *PLAUR* may decrease the number of Mast_cells_activated, thereby influencing the pathological processes of CTEPH.

To advance research in the drug treatment of CTEPH, our investigation has revealed the promising binding affinity of *HMOX1*-STANNSOPORFIN through targeted drug analysis and molecular docking studies.Notably, a study has demonstrated that STANNSOPORFIN effectively inhibits the proliferation and migration of the non-small cell lung cancer cell line A549^38^. Consequently, this suggests that STANNSOPORFIN has the potential to treat CTEPH by targeting *HMOX1* to inhibit pulmonary smooth muscle cell and endothelial cell migration and abnormal proliferation. However, the specific pathways through which this gene-targeting drug operates require further elucidation. To this end, we plan to design additional experiments that will not only substantiate the therapeutic effects of STANNSOPORFIN but also shed light on its molecular mechanisms of action. These studies will be crucial for validating the drug’s efficacy and safety in the context of CTEPH treatment.

In conclusion, the most important finding of this study is the first demonstration that Anoikis may be involved in the pathological process of CTEPH. The pathogenic mechanism of CTEPH is still unclear, and our findings will contribute to the study of the specific mechanism of CTEPH. CTEPH is a disease that is difficult to diagnose early and has a high mortality rate. Currently, there is a critical deficiency in biomarkers capable of predicting the severity and clinical outcomes of the disease. We found that *HMOX1* and *PLAUR* are two strong and promising diagnostic biomarkers for CTEPH, which could provide an easy way to diagnose CTEPH early in the clinic. Mast_cells_activated and Neutrophils may be involved in Anoikis resistance in CTEPH patients, offering novel insights into CTEPH therapeutic targets. STANNSOPORFIN emerges as a potential therapeutic agent targeting the Anoikis process in CTEPH, opening new horizons for treatment strategies.

The results of bioinformatics analysis and in vitro experiment provide a new direction for further study of CTEPH. Admittedly, we acknowledge the limitations of our study, including the limited data set and deeper in vivo validation. In order to minimize the bias caused by the limited data set, we used WGCNA, PPI, LASSO and SVM-RFE methods for multi-level exploration. Additionally, we also collected peripheral blood of CTEPH patients for PCR and WB in vitro experiment verification to reinforce the credibility of our findings. Looking ahead, we are actively collecting peripheral blood from CTEPH patients encountered in daily therapy for larger, more reliable studies. Concurrently, we are also developing a more reliable pulmonary artery ligation rat model to replace the traditional autothrombotic rat model, and will continue to investigate the specific mechanisms of the relevant genes in the future.

## Conclusions

In summary, the study demonstrates for the first time that Anoikis may be involved in the pathological process of CTEPH. *HMOX1* and *PLAUR* are two powerful and promising diagnostic biomarkers for CTEPH-related Anoikis. Mast_cells_activated and Neutrophils may be involved in Anoikis resistance in CTEPH patients, offering novel insights into CTEPH therapeutic targets. STANNSOPORFIN is a potential agents targeting Anoikis process therapy for CTEPH.

## Supplementary Information

Supplementary Information 1.

Supplementary Information 2.

Supplementary Information 3.

Supplementary Information 4.

Supplementary Information 5.

Supplementary Information 6.

Supplementary Information 7.

## Data Availability

The data used to support the findings of this study are available from the corresponding author.
