# Supplementary material for: Identification of Anoikis-related potential biomarkers and therapeutic drugs in chronic thromboembolic pulmonary hypertension via bioinformatics analysis and in vitro experiment
Source: Sci Rep. 2024 Dec 28;14:30663. doi: 10.1038/s41598-024-75251-1 (PMC11680802; doi:10.1038/s41598-024-75251-1)
Supplement: Supplementary file 1 — Supplementary Information 1. [file 41598_2024_75251_MOESM1_ESM.docx]

**Supplementary Material 1:R code**

1、limma

setwd("GSE130391")

library(tidyverse)

library(GEOquery)

gset = getGEO('GSE130391', destdir=".", AnnotGPL = F, getGPL = F)

class(gset)

gset[[1]]

pdata <- pData(gset[[1]])

table(pdata$source_name_ch1)

library(stringr)

group_list <- ifelse(str_detect(pdata$source_name_ch1, "CTEPH"), "CTEPHr",

"normal")

group_list = factor(group_list,

levels = c("normal","tumor"))

exp <- read.table("exp.txt",sep = "\t",row.names = 1,check.names = F,stringsAsFactors = F,header = T)

library(limma)

design=model.matrix(~group_list)

fit=lmFit(exp,design)

fit=eBayes(fit)

deg=topTable(fit,coef=2,number = Inf)

write.table(deg, file = "deg_all.txt",sep = "\t",row.names = T,col.names = NA,quote = F)

logFC=1

P.Value = 0.05

k1 = (deg$P.Value < P.Value)&(deg$logFC < -logFC)

k2 = (deg$P.Value < P.Value)&(deg$logFC > logFC)

deg$change = ifelse(k1,"down",ifelse(k2,"up","stable"))

table(deg$change)

1. WGCNA

install.packages("BiocManager")

BiocManager::install("preprocessCore")

BiocManager::install("impute")

install.packages("WGCNA")

library("tidyverse")

library("WGCNA")

setwd("WGCNA")

library(DESeq2)

counts_01A <- read.table("LIHC_counts_mRNA_01A.txt",sep = "\t",row.names = 1,check.names = F,stringsAsFactors = F,header = T)

exp <- read.table("LIHC_fpkm_mRNA_01A.txt", sep = "\t",row.names = 1,check.names = F,header = T)

com <- intersect(colnames(counts_01A),colnames(exp))

exp <- exp[,com]

counts_01A <- counts_01A[,com]

identical(colnames(counts_01A),colnames(exp))

gene <- "PDCD1"

med=median(as.numeric(exp[gene,]))

conditions=data.frame(sample=colnames(exp),

group=factor(ifelse(exp[gene,]>med,"high","low"),levels = c("low","high"))) %>%

column_to_rownames("sample")

dds <- DESeqDataSetFromMatrix(

countData = counts_01A,

colData = conditions,

design = ~ group)

dds <- DESeq(dds)

resultsNames(dds)

res <- results(dds)

save(res,file="res_deseq2_PDCD1.Rda")

DEG <- as.data.frame(res)%>%

arrange(padj) %>%

dplyr::filter(abs(log2FoldChange) > 1, padj < 0.05)

input <- exp[rownames(DEG),]

datExpr0 = as.data.frame(t(input))

gsg = goodSamplesGenes(datExpr0, verbose = 3)

gsg$allOK

if (!gsg$allOK){

# Optionally, print the gene and sample names that were removed:

if (sum(!gsg$goodGenes)>0)

printFlush(paste("Removing genes:", paste(names(datExpr0)[!gsg$goodGenes], collapse = ", ")));

if (sum(!gsg$goodSamples)>0)

printFlush(paste("Removing samples:", paste(rownames(datExpr0)[!gsg$goodSamples], collapse = ", ")));

# Remove the offending genes and samples from the data:

datExpr0 = datExpr0[gsg$goodSamples, gsg$goodGenes]

}

e = hclust(dist(datExpr0), method = "average")

par(cex = 0.6)

par(mar = c(0,4,2,0))

plot(sampleTree)

plot(sampleTree, main = "Sample clustering to detect outliers", sub="", xlab="", cex.lab = 1.5, cex.axis = 1.5, cex.main = 2)

abline(h = 67, col = "red")

clust = cutreeStatic(sampleTree, cutHeight = 67, minSize = 10)

table(clust)

keepSamples = (clust==1)

datExpr0 = datExpr0[keepSamples, ]

dev.off()

sampleTree2 = hclust(dist(datExpr0), method = "average")

plot(sampleTree2)

nGenes = ncol(datExpr0)

nSamples = nrow(datExpr0)

save(datExpr0, nGenes, nSamples,file = "Step01-WGCNA_input.Rda")

enableWGCNAThreads()

powers = c(1:20)

sft = pickSoftThreshold(datExpr0, powerVector = powers, verbose = 5)

par(mfrow = c(1,2))

cex1 = 0.9

plot(sft$fitIndices[,1], -sign(sft$fitIndices[,3])*sft$fitIndices[,2],

xlab="Soft Threshold (power)",ylab="Scale Free Topology Model Fit,signed R^2",type="n",

main = paste("Scale independence"));

text(sft$fitIndices[,1], -sign(sft$fitIndices[,3])*sft$fitIndices[,2],

labels=powers,cex=cex1,col="red");

abline(h=0.90,col="red")

plot(sft$fitIndices[,1], sft$fitIndices[,5],

xlab="Soft Threshold (power)",ylab="Mean Connectivity", type="n",

main = paste("Mean connectivity"))

text(sft$fitIndices[,1], sft$fitIndices[,5], labels=powers, cex=cex1,col="red")

sft

softPower =sft$powerEstimate

softPower = 2

adjacency = adjacency(datExpr0, power = softPower)

TOM = TOMsimilarity(adjacency)

dissTOM = 1-TOM

save(TOM,file = "TOM.Rda")

geneTree = hclust(as.dist(dissTOM), method = "average");

plot(geneTree, xlab="", sub="", main = "Gene clustering on TOM-based dissimilarity",

labels = FALSE, hang = 0.04)

minModuleSize = 30

dynamicMods = cutreeDynamic(dendro = geneTree, distM = dissTOM,

deepSplit = 2, pamRespectsDendro = FALSE,

minClusterSize = minModuleSize);

table(dynamicMods)

dynamicColors = labels2colors(dynamicMods)

table(dynamicColors)

plotDendroAndColors(geneTree, dynamicColors, "Dynamic Tree Cut",

dendroLabels = FALSE, hang = 0.03,

addGuide = TRUE, guideHang = 0.05,

main = "Gene dendrogram and module colors")

MEList = moduleEigengenes(datExpr0, colors = dynamicColors)

MEs = MEList$eigengenes

MEDiss = 1-cor(MEs);

METree = hclust(as.dist(MEDiss), method = "average")

plot(METree, main = "Clustering of module eigengenes",

xlab = "", sub = "")

MEDissThres = 0.1

abline(h=MEDissThres, col = "red")

merge = mergeCloseModules(datExpr0, dynamicColors, cutHeight = MEDissThres, verbose = 3)

mergedColors = merge$colors

mergedMEs = merge$newMEs

plotDendroAndColors(geneTree, mergedColors,"Dynamic Tree Cut",

dendroLabels = FALSE, hang = 0.03,

addGuide = TRUE, guideHang = 0.05,

main = "Gene dendrogram and module colors")

moduleColors = mergedColors

table(moduleColors)

colorOrder = c("grey", standardColors(50))

moduleLabels = match(moduleColors, colorOrder)-1

MEs = mergedMEs

dev.off()

clinical <- read.table("LIHC_fpkm_mRNA_01A_estimate_score.txt",sep = "\t",row.names = 1,check.names = F,stringsAsFactors = F,header = T)

clinical <- clinical[rownames(datExpr0),]

identical(rownames(clinical),rownames(datExpr0))

head(clinical)

datTraits = as.data.frame(do.call(cbind,lapply(clinical, as.numeric)))

rownames(datTraits) = rownames(clinical)

sampleTree2 = hclust(dist(datExpr0), method = "average")

traitColors = numbers2colors(datTraits, signed = FALSE)

plotDendroAndColors(sampleTree2,

traitColors,

groupLabels = names(datTraits),

main = "Sample dendrogram and trait heatmap")

dev.off()

MEs=orderMEs(MEs)

moduleTraitCor=cor(MEs, datTraits, use="p")

write.table(file="Step04-modPhysiological.cor.xls",moduleTraitCor,sep="\t",quote=F)

moduleTraitPvalue=corPvalueStudent(moduleTraitCor, nSamples)

write.table(file="Step04-modPhysiological.p.xls",moduleTraitPvalue,sep="\t",quote=F)

textMatrix=paste(signif(moduleTraitCor,2),"\n(",signif(moduleTraitPvalue,1),")",sep="")

dim(textMatrix)=dim(moduleTraitCor)

labeledHeatmap(Matrix=moduleTraitCor,

xLabels=colnames(datTraits),

yLabels=names(MEs),

ySymbols=names(MEs),

colorLabels=FALSE,

colors=blueWhiteRed(50),

textMatrix=textMatrix,

setStdMargins=FALSE,

cex.text=0.7,

cex.lab=0.7,

zlim=c(-1,1),

main=paste("Module-trait relationships"))

dev.off()

modNames = substring(names(MEs), 3)

geneModuleMembership = as.data.frame(cor(datExpr0, MEs, use = "p"))

a <- geneModuleMembership

a <- a %>% rownames_to_column()

MMPvalue = as.data.frame(corPvalueStudent(as.matrix(geneModuleMembership), nSamples))

names(geneModuleMembership) = paste("MM", modNames, sep="")

names(MMPvalue) = paste("p.MM", modNames, sep="")

traitNames=names(datTraits)

geneTraitSignificance = as.data.frame(cor(datExpr0, datTraits, use = "p"))

GSPvalue = as.data.frame(corPvalueStudent(as.matrix(geneTraitSignificance), nSamples))

names(geneTraitSignificance) = paste("GS.", traitNames, sep="")

names(GSPvalue) = paste("p.GS.", traitNames, sep="")

for (trait in traitNames){

traitColumn=match(trait,traitNames)

for (module in modNames){

column = match(module, modNames)

moduleGenes = moduleColors==module

if (nrow(geneModuleMembership[moduleGenes,]) > 1){

outPdf=paste(trait, "_", module,".pdf",sep="")

pdf(file=outPdf,width=7,height=7)

par(mfrow = c(1,1))

verboseScatterplot(abs(geneModuleMembership[moduleGenes, column]),

abs(geneTraitSignificance[moduleGenes, traitColumn]),

xlab = paste("Module Membership in", module, "module"),

ylab = paste("Gene significance for ",trait),

main = paste("Module membership vs. gene significance\n"),

cex.main = 1.2, cex.lab = 1.2, cex.axis = 1.2, col = module)

abline(v=0.8,h=0.5,col="red")

dev.off()

}

}

}

for (mod in 1:nrow(table(moduleColors)))

{

modules = names(table(moduleColors))[mod]

probes = colnames(datExpr0)

inModule = (moduleColors == modules)

modGenes = probes[inModule]

write.table(modGenes, file =paste0(modules,".txt"),sep="\t",row.names=F,col.names=F,quote=F)

}

3、Lasso

rm(list=ls())

library(glmnet)

library(readxl)

library(plyr)

library(caret)

library(ggplot2)

library(openxlsx)

library(Hmisc)

data<-read.xlsx("DEGs_exp1.xlsx")

x<-as.matrix(data[,-c(1:2)])

y<-as.double(data$fusta)

fit<-glmnet(x,y,family="binomial",nlambda=1000,alpha=1)

print(fit)

plot(fit,xvar="lambda")

lasso_fit<-cv.glmnet(x,y,family="binomial",alpha=1,type.measure="auc",nlambda=1000)

plot(lasso_fit)

print(lasso_fit)

lasso_best=glmnet（x=x,y=y,alpha=1,lambda=lasso_fit$lambda.min）

coef(lasso_best)

coefficient=coef(lasso_best,s=lasso_best$lambda.min)

coe=coefficient@x

coe=as.data.frame(coe)

Active_Index=which(as.numeric(coefficient)!=0)

active_coefficients=as.numeric(coefficient)[Active_Index]

variable=rownames(coefficient)[Active_Index]

variable=as.data.frame(variable)

variable=cbind(variable,coe)

4、SVM-RFE

install.packages("e1071")

install.packages("Hmisc")

install.packages("ggpubr")

install.packages("ROCR")

library(e1071)

library(Hmisc)

library(ggpubr)

library(ROCR)

setwd("GSE130391")

rt<-read.table("exp.txt",head=T,check.names = F,row.names = 1)

exp<-t(log(rt+1))

head(exp)[1:4,1:4]

cli<-read.table("cliData.txt",head=T,check.names = F,row.names = 1)

str(cli)

exp=exp %>% t() %>% as.data.frame()

set.seed(123)

index<-sample(nrow(cli),round(nrow(cli)*1))

trainee<-exp[index,]

traineeCli<-cli[index,]

library(e1071)

library(Hmisc)

library(ggpubr)

library(ROCR)

model=tune.svm(x=trainee,

y=traineeCli,

kernel="linear",

cost=c(0.001,0.01,0.1,1,5,10))

summary(model)

svm_model<-model$best.model

svm_train<-predict(svm_model,trainee)

result_train<-cbind(traineeCli,svm_train)

head(result_train)

#ROC

pred_train<-prediction(result_train[,2],result_train[,1])

ROC_train<-performance(pred_train,"tpr","fpr")

auc_train<-performance(pred_train,"auc")@y.values[[1]]

auc_train

plot(ROC_train,

col="red",

xlab="False postive rate",ylab="True positive rate",

lty=1,lwd=3,

main=paste("AUC",auc_train))

abline(0,1,lty=2,lwd=3)

5、corrplot

setwd("GSE130391")

mydata<-read.xlsx("corrplot.xlsx")

> summary(mydata)

res <- cor(mydata)

> install.packages("corrplot") > corrplot(res, method = "shade",shade.col = NA, tl.col ="black", tl.srt = 45, order = "AOE")

6、ggplot2

df<-read.csv("boxplot_example_1.csv",

header=T)

head(df)

library(ggplot2)

ggplot(data=df,aes(x=dose,y=len,fill=supp))+

geom_violin()+

geom_boxplot(width=0.2,

position = position_dodge(0.9))+

theme_bw()+

theme(panel.grid = element_blank())+

scale_fill_manual(values = c("yellowgreen", "violetred1"))

library(ggpubr)

ggplot(data=df,aes(x=dose,y=len,fill=supp))+

geom_violin()+

geom_boxplot(width=0.2,

position = position_dodge(0.9))+

theme_bw()+

theme(panel.grid = element_blank())+

scale_fill_manual(values = c("yellowgreen", "violetred1"))+

stat_compare_means(aes(group=supp))

ggplot(data=df,aes(x=dose,y=len,fill=supp))+

geom_violin()+

geom_boxplot(width=0.2,

position = position_dodge(0.9))+

theme_bw()+

theme(panel.grid = element_blank())+

scale_fill_manual(values = c("yellowgreen", "violetred1"))+

stat_compare_means(aes(group=supp),

method = "t.test")

ggplot(data=df,aes(x=dose,y=len,fill=supp))+

geom_violin()+

geom_boxplot(width=0.2,

position = position_dodge(0.9))+

theme_bw()+

theme(panel.grid = element_blank())+

scale_fill_manual(values = c("yellowgreen", "violetred1"))+

stat_compare_means(aes(group=supp),

method = "t.test",

label="p.signif")
