# Supplementary material for: Identification of Anoikis-related potential biomarkers and therapeutic drugs in chronic thromboembolic pulmonary hypertension via bioinformatics analysis and in vitro experiment
Source: Sci Rep. 2024 Dec 28;14:30663. doi: 10.1038/s41598-024-75251-1 (PMC11680802; doi:10.1038/s41598-024-75251-1)
Supplement: Supplementary file 7 — Supplementary Information 7. [file 41598_2024_75251_MOESM7_ESM.pdf]

Supplementary Material6:WB

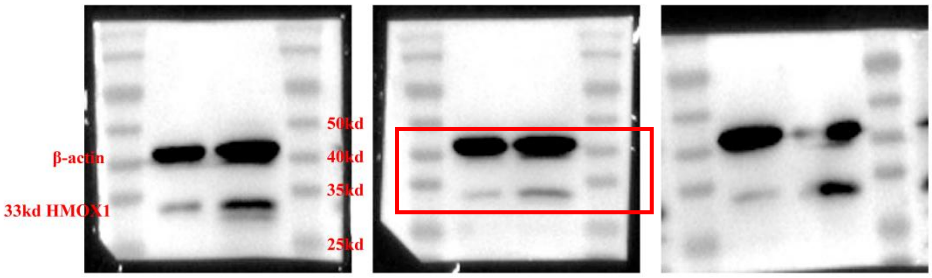

HMOX1

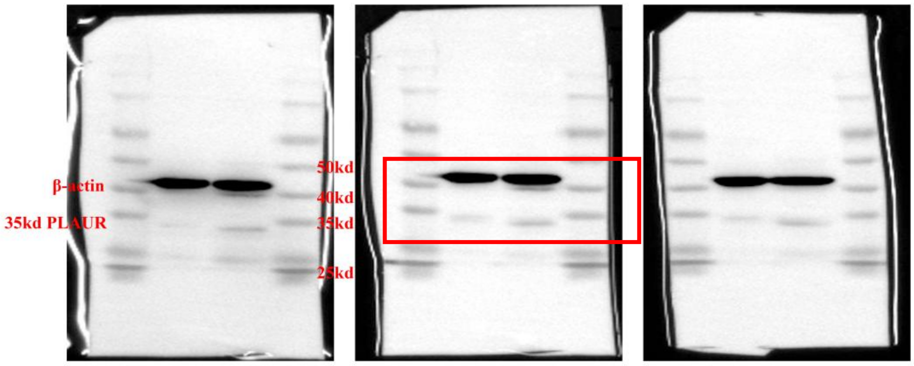

PLAUR

Note:The areas in red boxes are the representative figures in the manuscript.
